# Feasibility of Multiplex Cytokine Profiling in Preterm Labor: Towards Biomarker Discovery

**DOI:** 10.3390/biology14060714

**Published:** 2025-06-17

**Authors:** Ruth Llano, Inés Ardao, José Manuel Brea, Luz Romero, María P. Pata, Antón L. Martínez, Manuel Macía, María Isabel Loza

**Affiliations:** 1 Servicio de Obstetricia y Ginecología, Complejo Hospitalario Universitario de Santiago, 15706 Santiago de Compostela, Spain; 2 Health Research Institute of Santiago de Compostela (IDIS), 15706 Santiago de Compostela, Spainantonleandro.martinez@usc.es (A.L.M.); 3 Innopharma Drug Screening and Pharmacogenomics Platform, BioFarma Research Group, Center for Research in Molecular Medicine and Chronic Diseases (CiMUS), Department of Pharmacology, Pharmacy and Pharmaceutical Technology, University of Santiago de Compostela, 15706 Santiago de Compostela, Spain; 4 Kaertor Foundation, 15706 Santiago de Compostela, Spain; 5 Biostatech Advice, Training and Innovation in Biostatistics S.L., 15895 O Milladoiro, Spain; mariapata6@biostatech.com

**Keywords:** preterm delivery, urinary cytokines, cytokine network, inflammatory pathways, IL-1 signaling, GAMLSS, Luminex multiplex technology, exploratory biomarker discovery

## Abstract

Preterm delivery affects approximately 10% of pregnancies worldwide and remains a major clinical challenge due to the lack of reliable early predictive tools. Existing strategies are often invasive, relying on blood or amniotic fluid samples and requiring complex processing. In this study, we describe a novel non-invasive approach based on the multiplex detection of inflammatory cytokines in small urine volumes from pregnant women. To account for clinical and temporal variability, we applied Generalized Additive Models for Location, Scale, and Shape (GAMLSS) to adjust for gestational age at sampling and obstetric factors. Correlation network analyses revealed cytokine interactions that distinguished preterm from term deliveries, with macrophage-derived cytokines—MIP-1α, MIP-1β, IL-15, and IL-22—emerging as central nodes. These findings highlight the involvement of the IL-1 pathway in the pathophysiology of preterm labor. Furthermore, urinary IL-5 and IL-31 levels correlated positively with pregnancy duration, whereas IL-1β and IL-1Ra in urine and TNFα in amniotic fluid showed inverse associations. Altogether, this non-invasive methodology provides insight into immune dynamics during pregnancy and offers a foundation for future studies focused on biomarker discovery and mechanistic understanding of preterm birth.

## Simple Summary

Preterm birth, defined as giving birth before 37 weeks of pregnancy, affects about 10% of pregnancies around the world and is a major cause of illness and death in newborns. However, it remains difficult to identify which women are most at risk. Many current methods require taking samples of blood or fluid from around the baby, which are invasive and not ideal for routine use. In this study, we explored a new non-invasive method based on analyzing small urine samples from pregnant women. We measured inflammation-related molecules called cytokines, which play a key role in triggering labor. Using advanced statistical methods, we found specific patterns of cytokines in urine that were linked to how long the pregnancy lasted, and we identified a group of cytokines that may be especially important in early labor. These findings open the door to future studies and suggest that urine tests could eventually help monitor immune activity during pregnancy and support efforts to prevent preterm birth.

## 1. Introduction

Preterm delivery, defined as delivery occurring before the completion of 37 weeks of gestation (259 days), affects approximately 10% of all births globally, equating to an estimated 15 million premature infants each year [1]. Preterm delivery has significant clinical and societal implications, as it is the primary cause of hospitalization during pregnancy, the leading contributor to neonatal morbidity and mortality, and the most frequent cause of death among children under five, accompanied by substantial socioeconomic costs [2].

Preterm delivery is a complex, multifactorial condition with underlying mechanisms that remain incompletely elucidated. Most preterm delivery cases occur spontaneously and are frequently linked to inflammatory responses at the maternal–fetal interface [3]. Proposed pathophysiological mechanisms of preterm delivery include microbial-induced inflammation, immune intolerance between mother and fetus, decreased progesterone activity, and compromised decidual integrity—all contributing to immune activation associated with spontaneous preterm delivery [4]. This cascade prompts the release of cytokines and chemokines by immune cells within reproductive tissues. When these inflammatory pathways activate prematurely, they can initiate preterm labor, and if unresolved, may ultimately lead to preterm delivery [5].

Current therapeutic strategies for spontaneous preterm delivery primarily focus on inhibiting uterine contractions; however, these interventions generally delay delivery by only a few hours or days. Even though this brief delay can be critical, providing time for fetal lung maturation or for transferring the patient to a better-equipped healthcare facility, their effect on neonatal and perinatal mortality remains limited [6]. The modest efficacy of tocolytic drugs may stem from their action at later stages in the pathophysiological pathway of preterm labor [7]. These considerations reinforce the need for early identification of patients at risk of preterm delivery, which is the focus of the present study.

Despite the pressing need, early identification of women at risk for spontaneous preterm delivery remains a major challenge, particularly in low-risk populations where models based solely on maternal characteristics show limited predictive accuracy [8]. Various strategies have been explored, including transcriptomics, proteomics, metabolomics, lipidomic biomarkers, and circulating microparticles, aiming to uncover molecular signatures of risk [9,10,11,12,13]. While these approaches have advanced our mechanistic understanding of preterm birth, most rely on blood or amniotic fluid collection, and often require large sample volumes and complex processing, which limit their clinical utility. Moreover, none have yet demonstrated sufficient robustness or reproducibility for routine application.

Among the biomarker candidates under investigation, cytokines are particularly promising due to their central role in the inflammatory cascade that triggers both term and preterm labor [14,15]. However, cytokine concentrations are influenced by gestational age and obstetric factors, and their interplay is complex, often requiring multiplexed analyses rather than single-analyte approaches [16,17]. To overcome these limitations, non-invasive, high-sensitivity platforms capable of detecting multiple immune mediators in small sample volumes are essential for enabling clinically meaningful biomarker discovery. Since maternal immune activation is frequently systemic, urinary cytokines—originating predominantly from plasma—may serve as a non-invasive reflection of immunological processes during pregnancy, as previously demonstrated in other pathological conditions [18].

To study the activation of immunological pathways in clinical samples, highly sensitive measurements are essential, as some relevant immune proteins are present at low levels in maternal fluids [19]. Luminex multiplex technology, which uses fluorescence-labeled microspheres to detect multiple analytes simultaneously, offers distinct advantages over conventional ELISA, including higher throughput, reduced sample volume, and greater efficiency in multianalyte detection [20]. Compared to mass cytometry, which provides in-depth cellular phenotyping, Luminex is more accessible, requires less specialized infrastructure, and is better suited for soluble analyte quantification in translational research settings [21]. When applied to samples from pregnant women, this platform can capture subtle changes in immune signaling associated with preterm labor before clinical signs—such as uterine contractions or cervical alterations—become evident [15].

Our working hypothesis proposed that a non-invasive method for profiling inflammatory cytokines in urine samples from pregnant women could provide critical insights into the pathophysiology of preterm delivery and help to identify patients at risk of shorter pregnancies. To test this hypothesis, we developed and applied a non-invasive tailored methodology employing low urine volumes. To address the challenges posed by temporal and clinical variability in sample collection, we applied statistical adjustments to account for sampling timing and relevant clinical characteristics. Specifically, our objectives were to: (i) analyze correlations between cytokine levels in term and preterm deliveries to uncover mechanisms underlying preterm delivery; and (ii) investigate associations between cytokine concentrations and pregnancy duration.

## 2. Results

### 2.1. Descriptive Characteristics of Population

The demographic and obstetrical characteristics of the 79 patients included in the present study are summarized in Table 1 and Table 2.

The mean gestational age at sampling was 16 + 1 weeks (ranging from 14 + 5 to 18 + 0 weeks) in the AMN group, 30 + 2 weeks (ranging from 28 + 6 to 33 + 6 weeks) in the TPL group, and 39 + 0 weeks (ranging from 37 + 1 to 41 + 6 weeks) in the CSR group.

Five women delivered before 37 weeks of gestation: two women (5.9%) in the AMN group (31 + 1; 35 + 4 gestational weeks) and three women (18.8%) in the TPL group (26 + 1; 33 + 6; 36 + 5 gestational weeks) (Supplementary Figure S1), resulting in a total preterm delivery rate of 6.3%. Although consistent with general population estimates, this relatively low rate may limit the statistical power and generalizability of these findings.

The obstetrical characteristics of the three patient groups were largely comparable, with the exception of significant differences in parturition type (*p* < 0.05), the number of previous cesarean deliveries (*p* < 0.001), and the number of prior amniocenteses (*p* < 0.01). While this study was primarily exploratory and not powered to detect group differences in demographic or obstetric variables, the sample size was sufficient to enable robust multivariate modeling of cytokine levels across clinically defined subgroups. Importantly, these potential confounding differences were explicitly accounted for in downstream analyses, as described in Section 2.3.

### 2.2. MIP-1β, MIP-1α, IL-15, and IL-22 Are Identified as Relevant Cytokines for Preterm Delivery

**Figure 1:**
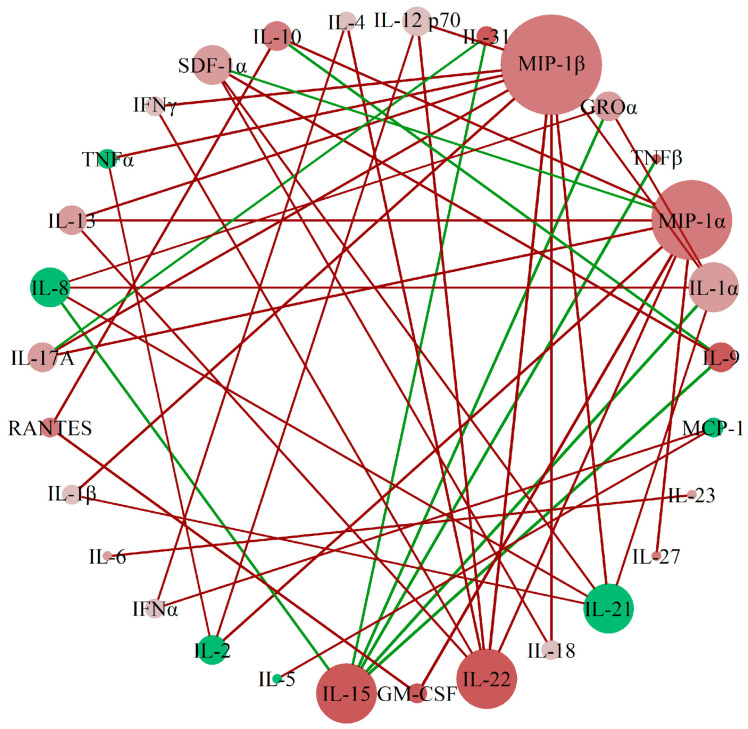
A network of cytokine interactions was established in urine samples of patients with preterm and term delivery. Network of perturbed cytokine concentration correlations between the group with preterm delivery (<37 weeks) and the group with term delivery (>37 weeks). Each node (sphere) represents one analyte; the node color indicates the direction of concentration change in the preterm group relative to the term group (red: increased; green: decreased), and color intensity reflects the magnitude of this change. Links (lines) connect cytokine pairs whose partial correlation differs significantly between groups: red links represent a gain of correlation in preterm vs. term delivery, while green links indicate a loss of correlation. Node size is proportional to the number of perturbed correlations involving that cytokine, i.e., the node’s degree in the network, reflecting its statistical centrality.

To assess the correlation patterns among cytokines between preterm and term gestations in urine samples, we applied perturbed correlation network analyses, adjusting for gestational age and body mass index, as detailed in Section 4.4.1. We identified a network of correlations whose principal nodes were MIP-1β, MIP-1α, IL-15, and IL-22. This prominence was based on the number and magnitude of perturbed correlations with other cytokines, as depicted in Figure 1. While centrality in this network highlights their statistical connectivity, it does not imply causal or biological primacy. However, given that these cytokines have been previously associated with macrophage activity and inflammation in pregnancy-related contexts, their centrality may warrant further investigation in future mechanistic studies.

### 2.3. Urine IL-1β, IL-1Ra, IL-31, and IL-5 Levels Are Associated with Duration of Pregnancy

To investigate the relationship between cytokine levels and pregnancy duration, we first assessed the potential influence of gestational age at sampling and obstetric variables that varied between groups as a result of clinical protocols (Table 2), as detailed in Section 4.4.2. These variables included parturition type, number of previous cesarean deliveries, and number of previous amniocenteses. This approach enabled us to identify and exclude cytokines whose levels were influenced by sampling timing or obstetric factors (Supplementary Table S1). As a result, the cytokines retained for further analysis—those unaffected by gestational age or obstetric variables—differed by fluid type. In amniotic fluid, these included GROα, IFNγ, IL-4, IL-18, MIP-1β, and TNFα; while in urine, GM-CSF, IL-1β, IL-1Ra, IL-5, IL-6, IL-8, IL-10, IL-22, IL-27, and IL-31 were selected.

**Figure 2:**
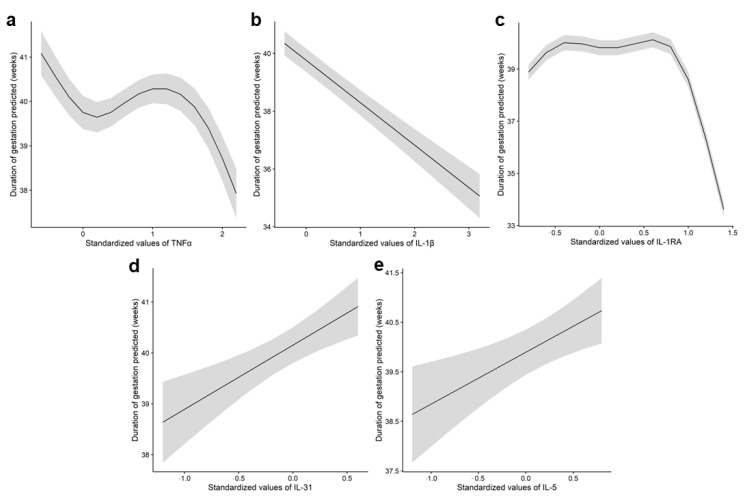
Association between TNFα in amniotic fluid and IL-1β, IL-1Ra, IL-31, and IL-5 in urine samples with pregnancy duration. Graph depicting the relationship between (**a**) amniotic fluid concentrations of TNFα and pregnancy duration, and relationship of urine concentrations of (**b**) IL-1β, (**c**) IL-1Ra, (**d**) IL-31, and (**e**) IL-5 with pregnancy duration. The line represents the marginal mean of the gestation duration as predicted by the GAMLSS model (μ), while the shaded area indicates the 95% confidence interval. Given the considerable variability observed in the cytokine data, the values were standardized to a uniform scale, with mean at 0 and standard deviation at 1, as previously described by Kuhn et al. (2013) [22].

Subsequently, we examined the correlation between cytokine levels and pregnancy duration (gestational age at delivery) using only the cytokines selected in the initial step. The strongest associations were observed in urine (Supplementary Table S2) (Figure 2), where IL-5 and IL-31 showed a positive correlation, indicating that elevated levels were associated with longer gestations, while IL-1β demonstrated an inverse relationship. Intermediate levels of IL-1Ra were not associated with gestational duration, as reflected by the flat slope of the marginal mean, while higher concentrations were associated with a noticeable decline in pregnancy duration. The final model selected for each cytokine, as well as the predictor η for model parameters mean (μ), variance (σ), skewness (ν), and kurtosis (τ) corresponding to TNFα in amniotic fluid and IL-1β, IL-1Ra, IL-31, and IL-5 in urine are presented in Supplementary Table S3.

## 3. Discussion

The main outcome of this study was the proposal of a non-invasive methodology for identifying cytokine signatures associated with preterm delivery. This approach addresses the inherent limitations of observational studies—particularly those related to sample availability, limited sample volume, time constraints for sample collection, and the obstetric characteristics of pregnant patients. It enabled: (i) the construction of a cytokine network to visualize interactions among cytokines in preterm versus term deliveries with MIP-1α, MIP-1β, IL-15, and IL-22 as central nodes, and (ii) the identification of associations between TNFα concentrations in amniotic fluid and IL-1β, IL-1Ra, IL-31, and IL-5 levels in urine with pregnancy duration. These findings offer valuable insights into the hypothesized pathophysiological mechanisms underlying preterm delivery, and lay the groundwork for innovative strategies to assess preterm delivery risk. Importantly, from a technological standpoint, this methodology paves the way for the integration of current high-throughput screening platforms into the development of prognostic assays for preterm delivery—an area with an urgent need for reliable predictive tools and therapeutic targets.

To reduce confounding effects, patients with conditions known to impact cytokine levels—such as autoimmune or infectious diseases, hypertension, immune disorders, or NSAID use—were excluded. The cohort was stratified by gestational stage to align with clinically relevant sampling points, resulting in three groups, as specified by clinical protocols: patients undergoing amniocentesis (15 to 17 + 6 weeks), those experiencing preterm labor (24 + 0 to 34 + 6 weeks), and those delivering at term (post-37 weeks).

To rigorously address the complexity and variability inherent to clinical cytokine data, we applied a statistical framework based on Generalized Additive Models for Location, Scale, and Shape (GAMLSS). This flexible modeling approach enabled adjustment for gestational age and obstetric factors, while capturing distributional properties beyond the mean—such as variance, skewness, and kurtosis [23]. Furthermore, we employed perturbed correlation network analysis to identify cytokine interactions that significantly differed between preterm and term deliveries.

Our analysis of perturbed correlations in urine cytokine concentrations identified MIP-1α, MIP-1β, IL-15, and IL-22 as central nodes in the cytokine network differentiating term from preterm gestations. While their statistical centrality suggests a potential role in the immune processes associated with preterm labor, it should be interpreted with caution, as centrality does not necessarily imply biological causality. These cytokines have been previously associated with macrophage activity and inflammatory signaling in pregnancy-related contexts, suggesting a plausible link to macrophage-driven responses. Future studies incorporating macrophage-specific markers or immunophenotyping would be required to confirm whether the observed cytokine profiles reflect a macrophage-dominant immune signature in preterm labor.

Specifically, MIP-1α expression in the decidua has been associated with preterm delivery, due to its contribution to the inflammatory milieu associated with preterm labor [24]. Although the role of MIP-1β in pregnancy is less well-characterized, it has been linked to hypertensive disorders, suggesting a broader impact of inflammatory pathways on gestational health [25]. IL-15, also produced by macrophages [26], is tightly regulated during pregnancy, with dysregulated expression correlating with adverse outcomes, such as miscarriage [27]. IL-22, a cytokine integral to the innate immune response and produced by macrophages [28], has been associated with both fetal injury and protection from bacterial infection [29].

The identification of cytokines produced by macrophages as central nodes in the correlations that distinguish term from preterm gestations underscores the importance of these cells in preterm delivery. Macrophages play key roles in several labor-related processes, including uterine changes preceding labor, cervical ripening and dilation, and the rupture of fetal membranes [5]. Moreover, an increase in activated macrophages has been observed in preterm delivery patients, suggesting that these cells may play a dual role: maintaining pregnancy through homeostatic mechanisms while also potentially inducing labor upon activation [4].

Apart from assessing correlations between cytokine concentrations in preterm and term pregnancy patients, we aimed to evaluate the relationship between cytokine levels and pregnancy duration. Given that sample collection followed clinical protocols, variations in gestational age at the time of sampling and other obstetric variables were introduced, potentially influencing cytokine concentrations [16]. To address this, we applied GAMLSS models to identify and exclude cytokines whose levels varied significantly with gestational age and obstetric variables such as the parturition type, number of prior cesarean sections, and number of previous amniocenteses.

Notably, the cytokines influenced by those factors differed between amniotic fluid and urine, reflecting their distinct origins—urine primarily derived from maternal plasma and amniotic fluid from both maternal and fetal sources [30]. It is important to note that urinary cytokine concentrations observed in our study cannot be attributed to urinary tract infections, as participants with infectious diseases were excluded by protocol. This strengthens the interpretation that urinary cytokine profiles reflect systemic immune activation rather than localized urinary tract inflammation.

This biological distinction highlights that different fluids may capture different aspects of the immunological landscape during pregnancy. While urine offers a minimally invasive and easily accessible matrix suitable for longitudinal monitoring and population-level screening [31], amniotic fluid may be more appropriate for detecting localized intrauterine inflammatory processes, particularly those involving the fetus or fetal membranes [30]. Consequently, the selection of the biological matrix should be aligned with the specific research or clinical objective—systemic surveillance versus localized diagnostics. Future studies might benefit from a multi-compartment approach, integrating data from urine, blood, and amniotic fluid to improve both the mechanistic understanding and the predictive accuracy of preterm birth biomarkers.

In this context, our analytical approach uncovered specific cytokine patterns in both urine and amniotic fluid that were associated with pregnancy duration, providing further evidence of the relevance of these fluids for preterm birth risk stratification. Elevated TNFα levels in amniotic fluid, along with high IL-1β and maximal IL-1Ra concentrations in urine, were associated with shorter pregnancies. In contrast, increased IL-31 and IL-5 levels in urine correlated with longer gestational duration. These findings support the inflammatory etiopathogenic hypothesis of preterm birth [32]. TNFα has been shown to promote prostaglandin synthesis, matrix metalloproteinase expression, and apoptosis in amnion cells—all processes closely linked to preterm labor [33,34]. Additionally, the observed negative correlations of IL-1β and IL-1Ra—the agonist and antagonist of the IL-1 receptor, respectively—with pregnancy duration highlight their potential role in preterm birth pathophysiology. Interestingly, IL-1 signaling is essential for endothelial permeability, which is a critical factor in preterm delivery pathogenesis [35].

**Figure 3:**
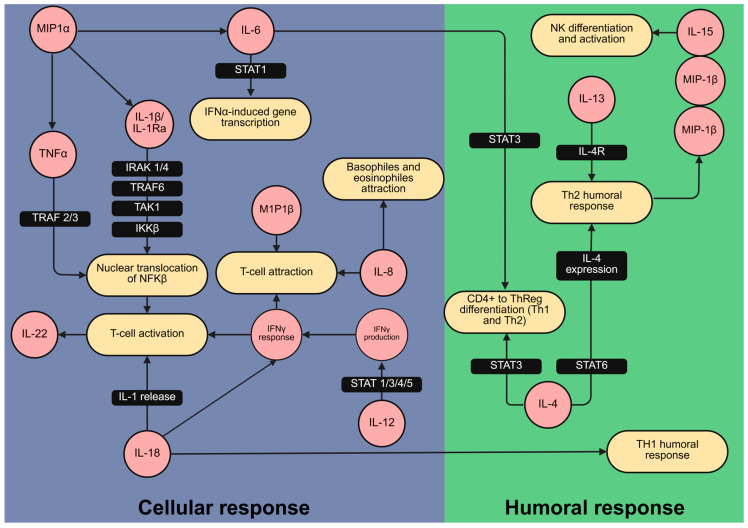
Cytokines related to cellular and humoral inflammatory responses varied between preterm delivery and at term delivery patients. Mapping of cytokines (in red) associated with the duration of pregnancy and likelihood of preterm delivery within the immunological signaling network. The network was created using the Reactome pathway database [38], which provides a curated collection of established signaling pathways and interactions. This mapping illustrates their involvement in cellular (blue) and humoral (green) immune responses relevant to preterm labor. Figure created with BioRender.com.

Building on this, the cytokine correlation patterns and their associations with gestational duration enabled us to identify key immune mediators involved in preterm delivery pathophysiology. In particular, macrophage-derived cytokines such as MIP-1α, MIP-1β, IL-15, and IL-22 appear to play interconnected roles in the inflammatory cascade leading to preterm labor. Upon activation by IL-1β [36], macrophages may contribute to the central role of the IL-1 pathway in the onset of preterm birth [37]. The observed changes in cytokine levels are consistent with signaling cascades in both innate and humoral immunity, as illustrated by the Reactome pathway database (Figure 3).

The methodology we developed addresses challenges inherent to clinical sampling—namely, invasiveness, limited sample volumes, and sampling heterogeneity—enabling the identification of cytokine interactions and their associations with pregnancy duration while minimizing bias. Translating these findings into clinical applications will require validation in larger patient cohorts, as well as prospective studies to establish standardized thresholds for cytokine concentrations, facilitating consistent risk assessment for preterm delivery. Moreover, this work contributes to understanding the mechanistic roles of the IL-1 pathway and macrophage-derived cytokines in the pathophysiology of preterm birth, with potential implications for therapeutic development. Beyond clinical translation, these results lay the groundwork for future research into high-throughput, immune-based strategies for early identification and prevention of preterm birth.

One limitation of this study is the relatively small number of patients who ultimately delivered preterm (n = 5), which may limit the statistical power of some findings and restrict the generalizability of our conclusions. While this study offers proof-of-concept evidence for the potential of urinary cytokines as predictive biomarkers, further validation in larger, prospective cohorts will be essential to confirm these observations and to develop clinically applicable prediction tools.

In summary, this study highlights the value of a non-invasive approach for cytokine detection in low urine volumes, combined with advanced statistical modeling to construct a cytokine network to distinguish preterm from term deliveries. Macrophage-derived cytokines—MIP-1α, MIP-1β, IL-15, and IL-22—were identified as central network nodes. Additionally, significant associations were found between TNFα levels in amniotic fluid and IL-1β, IL-1Ra, IL-31, and IL-5 in urine with pregnancy duration, particularly involving components of the IL-1 pathway. These findings contribute to a better understanding of the inflammatory mechanisms underlying preterm labor and offer a basis for future research aimed at developing predictive biomarkers and therapeutic interventions.

## 4. Materials and Methods

### 4.1. Study Description and Population

A prospective, observational single-center cohort study was conducted, with patient recruitment occurring between July 2014 and September 2016 at the Hospital Clínico Universitario de Santiago de Compostela (Galicia, Spain). All participants were pregnant women of legal age who provided voluntary informed consent. The study protocol and consent forms were approved by the Ethical Research Committee of the Health Areas of Santiago de Compostela and Lugo.

A total of 79 patients were enrolled in this study and categorized into three groups according to specific inclusion criteria (Table 3). The AMN (amniocentesis) group included patients who underwent amniocentesis for prenatal diagnosis between 14 + 5 and 18 weeks. The TPL (threatened preterm labor) group comprised patients who experienced preterm labor between 24 + 0 and 34 + 6 weeks, with identifiable uterine contractions and either a shortened cervical length (<25 mm up to 31 + 6 weeks or <15 mm at ≥32 weeks) or cervical dilation of at least 2 cm, even though they did not always progress to preterm delivery. Patients not eligible for uterine contraction inhibition were excluded from this group. The cesarean (CSR) group consisted of patients who underwent a scheduled cesarean section at term. The inclusion of these three groups was intended to capture specific time points for clinical sample collection as defined by clinical protocols throughout pregnancy [39,40], ensuring representative sampling across different gestational stages for the analysis of cytokine profiles.

To prevent immune-related confounding factors, we excluded patients with acute or chronic infectious diseases, as well as those receiving antibiotics or NSAIDs. Pregnant women with autoimmune disorders or those on immunosuppressive therapies were also excluded. To further reduce potential bias, patients with obstetric conditions, including premature rupture of membranes, hypertensive disorders of pregnancy, gestational diabetes, or multiple gestations, were not considered. Specific exclusion criteria for each group are detailed in Table 3.

### 4.2. Specimen Collection and Preparation

Amniotic fluid samples were collected when clinically indicated, either during amniocentesis (AMN and TPL groups) or at the time of cesarean delivery (CSR group). For cesarean deliveries, a Cornier pipelle (Gynétics, Lommel, Belgium) was used to obtain samples through hysterorrhaphy at the start of the surgical procedure. Since the cesarean sections were performed under neuraxial anesthesia administered immediately before sample collection, this timing is not expected to significantly alter cytokine concentrations [41]. Samples were kept at 4 °C for up to five hours between collection and processing. They were then centrifuged at 800× *g* for 10 min at 4 °C, aliquoted, and stored at −80 °C.

Urine samples were collected simultaneously with amniotic fluid samples by spontaneous micturition into sterile containers and stored at −80 °C. Although factors such as hydration, diet, or time of day may influence urinary cytokine concentrations, these variables were not controlled for, as the study was designed to reflect the conditions under which clinical samples are typically obtained.

### 4.3. Cytokine Analysis

The Cytokine & Chemokine 34-Plex Human ProcartaPlex Panel 1A immunoassay kit (EPX340-12167-901; Thermo Fisher, Alcobendas, Madrid, Spain) was used according to the manufacturer’s instructions to simultaneously detect and quantify 34 cytokines in urine and amniotic fluid samples, employing the dilution factors indicated by the manufacturer. The cytokines analyzed included eotaxin, GM-CSF, GROα, IFNα, IFNγ, IL-1β, IL-1α, IL-1Ra, IL-2, IL-4, IL-5, IL-6, IL-7, IL-8, IL-9, IL-10, IL-12 p70, IL-13, IL-15, IL-17A, IL-18, IL-21, IL-22, IL-23, IL-27, IL-31, IP-10, MCP-1, MIP-1α, MIP-1β, RANTES, SDF-1α, TNFα, and TNFβ. Plates were analyzed using a Luminex multiplexing instrument (MILLIPLEX^®^xMAP, Millipore, Burlington, MA, USA), and the level of each cytokine in each sample was quantified by interpolation in the corresponding calibration curve fitted to a four-parameter logistic regression model. Each amniotic fluid or urine sample was assayed in duplicate.

Cytokine values were standardized by subtracting the mean and dividing by the standard deviation, to get a distribution with mean at 0 and standard deviation of 1 (Supplementary Table S4). The obtained levels were then statistically analyzed to explore potential cytokine signatures related to preterm delivery.

### 4.4. Statistical Analysis

Due to the presence of non-linearity, dispersion, as well as heavy tailed distributions observed in the data, an extension of Generalized Additive Models (GAMs) [42,43], the Generalized Additive Models for Location, Scale and Shape (GAMLSS) [44], were selected, since these types of models allow us not only the inclusion of flexible additive effects or different family distributions, but also they allow us to model other parameters than the mean, such as the variance. Although these are more complex, they have been selected to enable improvements in the goodness of fit given the high variability in data.

All statistical analyses were conducted using R statistical software (version 4.0.2) employing the DGCA version 1.0.3 (Differential Gene Correlation Analysis) package for the partial correlation analysis and the gamlss version 5.1-7 (Generalized Additive Models for Location, Scale, and Shape) package for estimating the general additive models [42,45,46]. Given the considerable variability observed in the cytokine data, the values were standardized to a uniform scale, with mean at 0 and standard deviation at 1, as previously described by Kuhn et al. (2013) [22].

#### 4.4.1. Assessment of the Correlation Between Cytokine Levels in Preterm and Term Gestations

Perturbed correlations network analysis was conducted to determine whether the partial correlation of cytokine concentrations between each pair differed between preterm and term gestations. This analysis was adjusted for gestational age and body mass index, following the methodology described by Romero et al. (2015) [47]. Briefly, partial correlations were estimated using the method proposed by Sheskin (2003) [48], where differences in partial correlations between groups (perturbed correlations) were tested using Fisher’s z-transformation (to normalize the partial correlation distribution), with *p*-values adjusted by the Benjamini and Hochberg method [49]. A network was subsequently constructed by connecting cytokines (nodes) with significantly different correlations between groups. For each node, the mean absolute difference in correlations—quantifying the magnitude of correlation change between groups irrespective of direction—and the number of significantly perturbed correlations (network links) with other nodes were calculated.

#### 4.4.2. Assessment of the Influence of Gestational Age and Obstetric Features at the Time of Sample Collection

Before analyzing the relationship between cytokine levels and pregnancy duration, we evaluated the influence of gestational age at sample collection on cytokine levels, adjusting for obstetric covariates with significant group differences, as identified by the Kruskal–Wallis test, to account for clinical variability. The family distribution selected was the one with the lowest value of the Akaike information criterion (AIC) among the family of the continuous distributions (skew t type 3 distribution with an AIC of 555.04). Three models (full model, simple model, and null model) were considered for model selection. The full model incorporates both obstetric covariates and the potential flexible effect of the corresponding cytokine. In contrast, the simple model solely encompasses the potential flexible effect of the cytokine, while the null model represents just the intercept. The Likelihood Ratio Test was chosen for model selection because the models are nested. The cytokines for which the null model is selected are those considered not to be influenced by gestational age and are the candidates for the next step.

#### 4.4.3. Assessment of the Association Between Cytokine Levels and Duration of Pregnancy

After identifying those cytokines unaffected by gestational age and obstetric variables, an assessment was conducted to determine the association between them and the gestational age at the time of parturition (duration of pregnancy), with adjustments made for membership group (Group). The skew t type 4 family distribution was selected among the pool of continuous distributions since it was the one with the lowest AIC value (AIC of 248.94), and worm plots (a diagnostic tool for evaluating residuals) were utilized to verify the assumed distribution for the response. The four parameters of the selected distribution were modeled with identity link for the mean (µ), and with log link for the variance (σ), skewness (ν), and kurtosis (τ). Step-wise model selection with bidirectional elimination by AIC was employed. Penalized splines (P-splines) were selected for smooth functions [50], with effective degrees of freedom automatically selected by Generalized Cross-Validation [43].

For the association between biomarkers and duration of pregnancy, the model selection process implied the determination of a null model and a full model for each of the four parameters of the distribution and each cytokine. For µ parameter, the null model includes a linear effect of cytokine and group, and the full model includes a flexible effect of the cytokine, and an interaction term between cytokine and group (for assessing possible differences in the association between cytokine and time to parturition (related to group). For avoiding overparameterization, the full model for σ includes a flexible effect of the cytokine and a main effect of group; the full model for ν and τ only includes the linear effect of cytokine and the main effect of group. The null model for these three parameters includes only the intercept.

## 5. Conclusions

In this study, we demonstrate the feasibility of a non-invasive approach for cytokine profiling in pregnancy using small volumes of urine analyzed by multiplex Luminex technology. By applying advanced statistical models that adjust for gestational age and obstetric variables, we identified specific cytokine signatures associated with preterm delivery and pregnancy duration. Notably, macrophage-derived cytokines—MIP-1α, MIP-1β, IL-15, and IL-22—emerged as central components of the cytokine network distinguishing preterm from term deliveries, while urinary levels of IL-1β, IL-1Ra, IL-31, and IL-5 showed significant associations with gestational length.

These findings provide proof-of-concept evidence supporting the utility of urinary cytokine profiling as a minimally invasive tool for investigating immune mechanisms underlying preterm labor. Although validation in larger, prospective cohorts is required, our results establish a foundation for the development of immune-based biomarkers to improve early risk stratification and guide preventive strategies in obstetric care.

## Figures and Tables

**Table 1:** Demographic characteristics of the study population. Data are expressed as mean (standard deviation) for continuous variables and number (%) for categorical variables. AMN (amniocentesis); TPL (threatened preterm labor); CSR (cesarean).

Criteria	AMN Group (N = 34)	TPL Group (N = 16)	CSR Group (N = 29)
Age (years)	38.7 (2.7)	32.2 (3.7)	35.3 (4.8)
Weight (kg)	67.1 (10.2)	65.3 (7.6)	77.7 (13.5)
Height (m)	1.63 (0.06)	1.61 (0.03)	1.62 (0.07)
Body mass index (kg/m^2^)	25.4 (4.2)	25.2 (3.0)	29.8 (5.1)
Smoking patients (*n*)	5 (14.7)	2 (12.5)	4 (13.8)

**Table 2:** Obstetrical characteristics of the study population. Data are expressed as mean (standard deviation) for continuous variables and number (%) for categorical variables. The *p* value is based on Fisher’s exact test for categorical nominal variables and the linear trend test for categorical ordinal variables (alpha was set at 0.05; all tests are bilateral).

Obstetrical Characteristics	AMN Group (N = 34)	TPL Group (N = 16)	CSR Group (N = 29)	Total (N = 79)	*p* Value
Time to delivery (weeks)	23.2 (1.9)	7.1 (3.6)	0.0 (0.0)	-	
Amenorrhea at sampling (weeks)	16.2 (0.8)	30.3 (2.6)	39.0 (0.9)	-	
Amenorrhea at delivery (weeks)	39.5 (1.9)	37.5 (3.8)	39.0 (0.9)	-	
Number of previous gestations (*n*)					0.696
0	11 (32.4)	8 (50.0)	12 (41.4)	31 (39.2)	
1	13 (38.2)	7 (43.8)	9 (31.0)	29 (36.7)	
2	4 (11.8)	0 (0.0)	5 (17.2)	9 (11.4)	
3	3 (8.8)	1 (6.2)	2 (6.9)	6 (7.6)	
4	1 (2.9)	0 (0.0)	1 (3.4)	2 (2.5)	
5	2 (5.9)	0 (0.0)	0 (0.0)	2 (2.5)	
Parturition type I (*n*)					0.002
At term	26 (76.5)	13 (81.2)	0 (0.0)	39 (49.4)	
Preterm	2 (5.9)	3 (18.8)	0 (0.0)	5 (6.3)	
Cesarean	6 (17.6)	0 (0.0)	29 (100.0)	35 (44.3)	
Parturition type II (*n*)					0.038
Nonpremature	32 (94.1)	13 (81.2)	29 (100.0)	74 (93.7)	
Premature	2 (5.9)	3 (18.8)	0 (0.0)	5 (6.3)	
Number of previous parturitions (*n*)					0.076
0	16 (47.1)	9 (56.2)	25 (86.2)	50 (63.3)	
1	14 (41.2)	6 (37.5)	3 (10.3)	23 (29.1)	
2	3 (8.8)	1 (6.2)	1 (3.4)	5 (6.3)	
3	1 (2.9)	0 (0.0)	0 (0.0)	1 (1.3)	
Type of previous parturitions (*n*)					0.453
At term	19 (95.0)	6 (85.7)	13 (86.7)	38 (90.5)	
Preterm	1 (5.0)	1 (14.3)	2 (13.3)	4 (9.5)	
Number of previous cesarean deliveries (*n*)					0.009
0	31 (91.2)	16 (100.0)	18 (62.1)	65 (82.3)	
1	2 (5.9)	0 (0.0)	9 (31.0)	11 (13.9)	
2	1 (2.9)	0 (0.0)	2 (6.9)	3 (3.8)	
Number of previous abortions (excluding induced abortions) (*n*)					0.669
0	23 (67.6)	14 (87.5)	24 (82.8)	61 (77.2)	
1	8 (23.5)	2 (12.5)	3 (10.3)	13 (16.5)	
2	1 (2.9)	0 (0.0)	1 (3.4)	2 (2.5)	
3	2 (5.9)	0 (0.0)	1 (3.4)	3 (1.3)	
Number of induced abortions (*n*)					0.685
0	33 (97.1)	16 (100.0)	27 (93.1)	76 (96.2)	
1	1 (2.9)	0 (0.0)	1 (3.4)	2 (2.5)	
3	0 (0.0)	0 (0.0)	1 (3.4)	1 (1.3)	
Type of previous abortions (*n*)					0.371
Early	10 (90.9)	1 (50.0)	5 (71.4)	16 (80.0)	
Late	1 (9.1)	1 (50.0)	2 (28.6)	4 (20.0)	
Number of uterine curettages (*n*)					0.928
0	28 (82.4)	14 (87.5)	24 (82.8)	66 (83.5)	
1	4 (11.8)	2 (12.5)	4 (13.8)	10 (12.7)	
2	1 (2.9)	0 (0.0)	0 (0.0)	1 (1.3)	
3	1 (2.9)	0 (0.0)	1 (3.4)	2 (2.5)	
Number of previous amniocenteses (*n*)					0.002
0	0 (0.0)	14 (87.5)	26 (89.7)	40 (50.6)	
1	34 (100.0)	2 (12.5)	3 (10.3)	39 (49.4)	

**Table 3:** Inclusion and exclusion criteria for the study population. AMN (amniocentesis); TPL (threatened preterm labor); CSR (cesarean).

	AMN Group (N = 34)	TPL Group (N = 16)	CSR Group (N = 29)
Inclusion criteria			
Common to all groups	Pregnant women of legal age Expressed voluntary and informed consent		
Category-specific			
Patient stratification	Patients subjected to amniocentesis for prenatal diagnosis	Patients diagnosed with threatened preterm labor	Patients who underwent cesarean delivery at term
Gestational week range	14 + 5 to 18	24 + 0 to 34 + 6	37 + 0 to 42 + 0
Exclusion criteria			
Common to all groups	Patients carrying infectious or autoimmune diseases Patients being treated with corticoids, immunosuppressants or NSAIDs Hypertensive states of pregnancy Treatment with antimicrobial drugs Feverish condition Gestational diabetes Twin gestations		
Category-specific			
Patient conditions at the time of sample collection		Premature rupture of membranes Not eligible for uterine contraction inhibition treatment	Premature rupture of membranes Labor
Obstetric conditions at the time of sample collection	Fetal chromosomal alterations Fetal malformations	Mullerian malformations Hydramnios Known cervical incompetence	

## Data Availability

The data that support the findings of this study are available from the corresponding authors upon request.

## Supplementary Materials

The following supporting information can be downloaded at: https://www.mdpi.com/article/10.3390/biology14060714/s1, Table S1: Association of cytokines with gestational age and obstetric variables; Table S2: GAMLSS parameter estimates for the association of cytokines with duration of gestation; Table S3: Predictor η f for model parameters mean (μ), variance (σ), skewness (ν), and kurtosis (τ) in the five selected cytokines. Table S4: Descriptive statistics of standardized cytokine levels in urine and amniotic fluid across study groups. Figure S1: Violin plot with boxplot overlay showing the distribution of gestational duration across groups.

## Abbreviations

The following abbreviations are used in this manuscript:

GAMLSS	Generalized Additive Models for Location, Scale, and Shape
GAM	Generalized Additive Models
AIC	Akaike Information Criterion
DGCA	Differential Gene Correlation Analysis
AMN	Amniocentesis
TPL	Threatened Preterm Labor
CSR	Cesarean
