# Supplementary material for: Feasibility of Multiplex Cytokine Profiling in Preterm Labor: Towards Biomarker Discovery
Source: Biology (Basel). 2025 Jun 17;14(6):714. doi: 10.3390/biology14060714 (PMC12189677; doi:10.3390/biology14060714)
Supplement: Supplementary file 1 [file biology-14-00714-s001.zip › biology-3656817-supplementary.pdf]

Feasibility of Multiplex Cytokine Profiling in Preterm Labor: Towards Biomarker Discovery

Ruth Llano, Inés Ardao, José Manuel Brea, Luz Romero, María P. Pata, Antón L. Martínez, Manuel Macía and María Isabel Loza

Table S1. **Association of cytokines with gestational age and obstetric variables**. The range, mean, and standard deviation (SD) as well as the effective degrees of freedom (edf); Chi-squared statistic from ANOVA table (χ^2) and significance are shown.

Cytokines	Amniotic fluid						Urine					
	Range (pg/ml)	Mean (pg/ml)	SD	edf	χ^2	Significance[a]	Range (pg/ml)	Mean (pg/ml)	SD	edf	χ^2	Significance[a]
**MIP-1α**	21-477	148.8	80.0	2.646	48.495	***	20-421	158.0	86.1	1.760	9.231	**
**SDF-1α**	22-1963	715.7	270.1	3.478	82.052	***	22-1840	1037.6	428.1	1.000	7.717	**
**IL-27**	4-106	7.8	2.6	2.534	58.491	***	4-40.5	6.9	1.3	1.337	2.183	ns
**IL-1β**	4-794.5	21.7	66.7	2.929	25.669	***	4-630.5	21.8	50.5	1.946	4.306	ns
**IL-2**	5-64.50	24.4	16.2	1.000	7.805	**	5-81	7.3	1.6	2.147	9.598	*
**IL-4**	10-259.5	84.5	61.2	1.141	0.094	ns	10-285	19.8	7.0	2.901	12.744	**
**IL-5**	5-80.5	20.5	13.5	2.736	53.994	***	5-67	24.4	5.0	1.002	2.128	ns
**IP-10**	6-3051	916.2	909.8	7.022	1775.880	***	5-4430	95.0	119.1	1.000	4.948	*
**IL-6**	4-6649	884.6	1268.8	2.426	12.588	**	5-6693	18.5	16.2	1.968	6.257	ns
**IL-7**	4-98	24.7	13.1	2.360	37.386	***	4-73	38.6	25.7	2.960	9.053	*
**IL-8**	27.5-5677.5	1023.1	1155.2	4.151	222.032	***	27-3676	368.5	509.5	1.825	2.897	ns
**IL-10**	5-44	12.7	5.5	4.842	222.932	***	6-28	9.6	1.3	1.000	0.113	ns
**Eotaxin**	3-197	13.9	7.7	3.213	19.635	***	4-58	10.6	7.5	3.871	36.819	***
**IL-12p70**	5-79.5	10.9	4.5	1.979	29.155	***	5-133	10.1	3.9	3.100	12.316	**

**IL-13**	5-114	9.9	2.7	1.000	3.95	*	5-126	9.4	3.7	3.554	22.786	***
**IL-17A**	4-96.5	8.8	4.5	5.793	368.663	***	5-144	6.9	1.5	1.011	4.456	*
**IL.31**	10-81.5	20.4	6.0	5.092	170.005	***	12-68	26.0	3.7	1.000	0.145	ns
**IL-1RA**	5-7966	501.6	1063.2	4.373	257.473	***	5-7710	1516.7	1026.6	2.064	4.954	ns
**RANTES**	22-6006	190.7	215.2	3.689	155.449	***	20-5574.5	366.9	956.0	2.276	49.996	***
**IFNγ**	6-347	12.9	4.1	1.919	1.958	ns	6-849	40.4	33.5	3.379	18.684	***
**GM-CSF**	5-92	15.8	8.7	2.349	12.341	**	6-45	7.5	1.1	1.000	0.678	ns
**TNFα**	6-68.5	21.7	12.1	1.959	5.600	ns	6-195	12.2	5.7	3.404	17.450	**
**MIP-1β**	50-1598.5	797.3	326.1	1.000	1.150	ns	40-2144	684.6	285.2	1.719	8.581	*
**IFNα**	7-481.5	13.7	4.9	3.000	43.025	***	8-54	17.9	3.0	3.549	29.129	***
**MCP-1**	8-15795.5	2725.1	2534.5	6.144	1567.664	***	8-19300.5	8189.9	5340.7	1.740	20.21	***
**IL-9**	6-464.5	19.5	9.9	6.799	358.420	***	7-49	32.4	6.5	1.000	47.78	***
**TNFβ**	10-598	32.4	17.2	5.339	499.890	***	10-129	63.7	13.5	3.122	41.283	***
**GROα**	16-4006	245.4	254.3	2.359	8.042	ns	16-1291.5	34.8	36.7	1.949	16.520	***
**IL-1α**	17-1616	77.0	54.2	4.152	125.221	***	20-972	87.1	48.7	2.694	17.116	***
**IL-23**	8-83	17.7	4.6	3.134	43.316	***	9-89	23.8	21.9	1.000	10.23	***
**IL-15**	7-334	11.6	3.3	2.214	40.805	***	7-39	15.8	17.6	1.124	4.01	*
**IL-18**	10-2811	151.6	184.3	2.145	5.321	ns	17-8545	356.4	336.0	2.317	9.567	*
**IL-21**	7-178	23.4	11.0	2.130	39.876	***	9-59	26.9	12.3	1.379	8.515	**
**IL-22**	8-31	16.2	4.8	1.793	15.953	***	10-30	19.7	7.4	1.000	0.395	ns

[a] ***: p-value ≤ 0.001; **: 0.001 < p-value ≤ 0.01; *: 0.01 < p-value ≤ 0.05; ns: p-value > 0.05; Chi-squared test.

Table S2. GAMLSS parameter estimates for the association of cytokines with duration of gestation. Parametric coefficients for mean (μ), variance (σ), skewness (ν), and kurtosis (τ) are expressed as intercept, estimated cytokine effect, and contrast between groups, lower (LCI) and upper (UCI) limits of 95% confidence interval; standard error (SE), t-statistic, and p-value are shown. The presence of flexibility in the parameter estimation is denoted by the term s (smooth).

Cytokine		Variable	Estimate (Beta)	LCI	UCI	SE	t statistic	p value
		**Amniotic fluid**						
**TNFα in amniotic fluid**	μ	Intercept	40.281	39.910	40.647	0.187	215.48	≤ 0.001
		s(TNFα)	-0.677	-0.975	-0.380	0.152	-4.460	≤ 0.001
		TPL group vs AMN group	-2.521	-3.466	-1.577	0.482	-5.231	≤ 0.001
		CSR group vs AMN group	-0.874	-1.300	-0.448	0.217	-4.020	≤ 0.001
		TPL group vs CSR group	-1.647	-2.548	-0.746	0.460	-3.583	0.001
	σ	Intercept	-0.260	-0.522	0.002	0.134	1.946	0.057
		s(TNFα)	-0.158	-0.518	0.203	0.184	-0.855	0.396
	ν	Intercept	0.279	-0.292	0.850	0.291	0.957	0.342
	τ	Intercept	12.525	-677.820	702.871	352.224	0.036	0.972
		**Urine**						
**IL1 β in urine**	μ	Intercept	39.772	39.383	40.161	0.199	200.246	≤ 0.001
		s(IL-1β)	-1.404	-1.548	-1.261	0.073	-19.134	≤ 0.001
		TPL group vs AMN group	-1.153	-1.860	-0.447	0.360	-3.200	0.002
		CSR group vs AMN group	-1.203	-1.739	-0.667	0.274	-4.396	≤ 0.001
		TPL group vs CSR group	0.052	-0.683	0.787	0.375	0.138	0.890
	σ	Intercept	-0.265	-0.568	0.038	0.155	-1.714	0.091
		s(IL-1β)	-0.641	-1.034	-0.248	0.201	-3.196	0.002
	ν	Intercept	0.573	-0.070	1.217	0.328	1.746	0.085
	τ	Intercept	2.433	-0.620	5.486	1.558	1.562	0.123
**IL-1Ra in urine**	μ	Intercept	38.757	38.451	39.064	0.156	247.80	≤ 0.001
		s(IL-1RA)	-2.945	-2.945	-2.945	0.000	-32.223	≤ 0.001
		TPL group vs AMN group	-0.866	-1.173	-0.560	0.156	-5.538	≤ 0.001
		CSR group vs AMN group	-0.697	-1.161	-0.233	0.237	-2.944	0.005
		TPL group vs CSR group	-0.170	-0.495	0.155	0.166	-1.027	0.308
	σ	Intercept	-1.063	-1.385	-0.741	0.164	-6.467	≤ 0.001
		s(IL-1RA)	-3.697	-4.255	-3.139	0.285	-12.985	≤ 0.001
		TPL group vs AMN group	0.083	-0.483	0.650	0.289	0.288	0.774

		CSR group vs AMN group	-0.083	-0.533	0.367	0.229	-0.361	0.719
		TPL group vs CSR group	0.146	-0.425	0.717	0.292	0.501	0.618
	ν	Intercept	0.656	0.153	1.160	0.257	2.553	0.013
	τ	Intercept	3.721	3.590	3.853	0.067	55.53	≤ 0.001
**IL-31 in urine**	μ	Intercept	40.153	39.808	40.498	0.176	228.01	≤ 0.001
		s(IL-31)	1.262	0.627	1.897	0.324	3.895	≤ 0.001
		TPL group vs AMN group	-1.162	-1.768	-0.555	0.309	-3.756	≤ 0.001
		CSR group vs AMN group	-1.101	-1.522	-0.679	0.215	-5.1133	≤ 0.001
		TPL group vs CSR group	-0.061	-0.625	0.503	0.288	-0.211	0.833
	σ	Intercept	-0.311	-0.738	0.117	0.218	-1.425	0.159
		TPL group vs AMN group	0.133	-0.600	0.866	0.374	0.355	0.723
		CSR group vs AMN group	-0.354	-0.918	0.210	0.288	-1.229	0.223
		TPL group vs CSR group	0.487	-0.254	1.228	0.378	1.288	0.202
	ν	Intercept	0.236	-0.339	0.811	0.293	0.804	0.424
	τ	Intercept	1.421	0.289	2.554	0.578	2.460	0.016
**IL-5 in urine**	μ	Intercept	9.894	39.445	40.342	0.229	174.49	≤ 0.001
		s(IL-5)	1.050	0.393	1.706	0.335	3.132	0.003
		TPL group vs AMN group	-0.799	-1.582	-0.017	0.399	-2.002	0.049
		CSR group vs AMN group	-1.067	-1.569	-0.565	0.256	-4.163	≤ 0.001
		TPL group vs CSR group	0.268	-0.447	0.982	0.365	0.734	0.465
	σ	Intercept	-0.093	-0.460	0.274	0.187	-0.496	0.621
		TPL group vs AMN group	-0.067	-0.791	0.656	0.369	-0.183	0.856
		CSR group vs AMN group	-0.641	-1.240	-0.041	0.306	-2.094	0.040
		TPL group vs CSR group	0.573	-0.203	1.350	0.396	1.447	0.153
	ν	Intercept	0.317	-0.282	0.916	0.306	1.037	0.303
	τ	Intercept	1.507	0.446	2.567	0.541	2.785	0.007

Table S3. Predictor η f for model parameters mean (μ), variance (σ), skewness (ν), and kurtosis (τ) in the five selected cytokines. γ_0 represents the intercept, γ_1(ifGroup=TPL) + γ_2(ifGroup=CSR) is the main effect of factor Group (with AMN as reference category), γ_3(cytokine X) are linear additive terms of cytokine X, and s_0(cytokine X) are smoothing additive terms for cytokine X.

**TNFα in amniotic fluid**	$\eta_\mu = \gamma_0 + \gamma_1$(ifGroup=TPL)+ γ_2(ifGroup=CSR) + s_0(TNFα)
	$\eta_\sigma = \gamma_0 + s_0$(TNF$\alpha$)
	$\eta_\nu = \gamma_0{}^a$
	$\eta_\tau = \gamma_0{}^a$
**IL-1β in urine**	$\eta_\mu = \gamma_0 + \gamma_1$(ifGroup=TPL) + γ_2(ifGroup=CSR) + γ_3(IL1β)
	$\eta_\sigma = \gamma_0 + s_0$(IL1β)
	$\eta_\nu = \gamma_0{}^a$
	$\eta_\tau = \gamma_0{}^a$
**IL-1Ra in urine**	$\eta_\mu = \gamma_0 + \gamma_1$(ifGroup=TPL) + γ_2(ifGroup=CSR) + s_0(IL1.RA)
	$\eta_\sigma = \gamma_0 + \gamma_1$(ifGroup=TPL) + γ_2(ifGroup=CSR) + s_0(IL1.RA)
	$\eta_\nu = \gamma_0{}^a$
	$\eta_\tau = \gamma_0{}^a$
**IL-5 in urine**	$\eta_\mu = \gamma_0 + \gamma_1$(ifGroup=TPL) + γ_2(ifGroup=CSR) + γ_3(IL5)
	$\eta_\sigma = \gamma_0 + \gamma_1$(ifGroup=2) + γ_2(ifGroup=3)
	$\eta_\nu = \gamma_0{}^a$
	$\eta_\tau = \gamma_0{}^a$
**IL-31 in urine**	$\eta_\mu = \gamma_0 + \gamma_1$(ifGroup=TPL) + γ_2(ifGroup=CSR) + γ_3(IL31)
	$\eta_\sigma = \gamma_0 + \gamma_1$(ifGroup=TPL) + γ_2(ifGroup=CSR)
	$\eta_\nu = \gamma_0{}^a$
	$\eta_\tau = \gamma_0{}^a$

[a]For parameters skewness and kurtosis, the predictor only includes the intercept.

Table S4. Descriptive statistics of standardized cytokine levels in urine and amniotic fluid across study groups. Mean (standard deviation) and range of cytokine concentrations, expressed as standardized values (mean = 0, SD = 1), for each study group: amniocentesis (AMN), threatened preterm labor (TPL), and cesarean section at term (CSR). Data are presented separately for urine (left panel) and amniotic fluid (right panel). The total number of valid samples for each cytokine is indicated as "N".

**Urine**				
	**AMN (N=33)**	**TPL (N=16)**	**CSR (N=29)**	**Total (N=78)**
**MIP-1α**				
N	33	16	29	78
Mean (SD)	0.03 (0.52)	0.55 (0.83)	0.42 (0.52)	0.28 (0.63)
Range	-0.70 - 1.10	-0.75 - 2.45	-0.53 - 1.84	-0.75 - 2.45
**SDF-1α**				
N	33	16	29	78
Mean (SD)	0.79 (0.94)	1.19 (1.07)	1.38 (0.73)	1.09 (0.93)
Range	-0.75 - 2.74	-0.61 - 2.75	-0.34 - 2.85	-0.75 - 2.85
**IL-27**				
N	33	16	29	78
Mean (SD)	-0.32 (0.07)	-0.27 (0.12)	-0.32 (0.05)	-0.31 (0.08)
Range	-0.44 - -0.14	-0.44 - 0.12	-0.37 - -0.24	-0.44 - 0.12
**IL-1β**				
N	33	16	29	78
Mean (SD)	-0.23 (0.16)	-0.01 (0.89)	-0.31 (0.03)	-0.21 (0.42)
Range	-0.33 - 0.25	-0.33 - 3.23	-0.35 - -0.21	-0.35 - 3.23
**IL-2**				
N	33	16	29	78
Mean (SD)	-0.50 (0.07)	-0.43 (0.20)	-0.51 (0.07)	-0.49 (0.11)
Range	-0.58 - -0.27	-0.65 - 0.19	-0.65 - -0.39	-0.65 - 0.19
**IL-4**				
N	33	16	29	78
Mean (SD)	-0.48 (0.17)	-0.43 (0.14)	-0.40 (0.18)	-0.44 (0.17)
Range	-0.66 - 0.11	-0.59 - -0.04	-0.64 - 0.36	-0.66 - 0.36
**IL-5**				
N	33	16	29	78
Mean (SD)	0.10 (0.29)	-0.06 (0.54)	0.23 (0.28)	0.11 (0.36)
Range	-0.40 - 0.77	-1.18 - 0.77	-0.32 - 0.77	-1.18 - 0.77
**IP-10**				
N	33	16	29	78
Mean (SD)	-0.41 (0.12)	-0.45 (0.06)	-0.29 (0.26)	-0.38 (0.19)
Range	-0.52 - -0.05	-0.52 - -0.30	-0.52 - 0.68	-0.52 - 0.68
**IL-6**				
N	33	16	29	78
Mean (SD)	-0.30 (0.02)	-0.29 (0.03)	-0.29 (0.03)	-0.30 (0.03)
Range	-0.32 - -0.23	-0.32 - -0.17	-0.31 - -0.21	-0.32 - -0.17
**IL-7**				
N	33	16	29	78
Mean (SD)	0.76 (1.49)	0.51 (0.86)	1.33 (1.39)	0.92 (1.37)
Range	-0.72 - 5.88	-0.43 - 2.33	-0.67 - 6.91	-0.72 - 6.91
**IL-8**				

N	33	16	29	78
Mean (SD)	-0.33 (0.44)	-0.12 (0.75)	-0.50 (0.27)	-0.35 (0.49)
Range	-0.66 - 1.45	-0.67 - 1.93	-0.65 - 0.84	-0.67 - 1.93
**IL-10**				
N	33	16	29	78
Mean (SD)	-0.42 (0.25)	-0.35 (0.27)	-0.38 (0.28)	-0.39 (0.26)
Range	-0.82 - 0.12	-0.72 - 0.12	-0.93 - 0.22	-0.93 - 0.22
**Eotaxin**				
N	33	16	29	78
Mean (SD)	-0.44 (0.10)	-0.26 (0.40)	-0.38 (0.36)	-0.38 (0.29)
Range	-0.60 - -0.22	-0.58 - 1.04	-0.58 - 1.43	-0.60 - 1.43
**IL-12 p70**				
N	33	16	29	78
Mean (SD)	-0.33 (0.25)	-0.21 (0.25)	-0.20 (0.31)	-0.26 (0.28)
Range	-0.55 - 0.60	-0.49 - 0.34	-0.55 - 1.11	-0.55 - 1.11
**IL-13**				
N	33	16	29	78
Mean (SD)	-0.36 (0.22)	-0.27 (0.19)	-0.23 (0.26)	-0.29 (0.23)
Range	-0.55 - 0.41	-0.49 - 0.19	-0.49 - 0.86	-0.55 - 0.86
**IL-17A**				
N	33	16	29	78
Mean (SD)	-0.32 (0.09)	-0.27 (0.19)	-0.33 (0.07)	-0.31 (0.11)
Range	-0.46 - -0.14	-0.46 - 0.37	-0.46 - -0.22	-0.46 - 0.37
**IL-31**				
N	33	16	29	78
Mean (SD)	-0.04 (0.26)	-0.10 (0.43)	-0.02 (0.22)	-0.05 (0.29)
Range	-0.45 - 0.51	-1.04 - 0.46	-0.45 - 0.38	-1.04 - 0.51
**IL-1RA**				
N	33	16	29	78
Mean (SD)	-0.09 (0.36)	-0.17 (0.55)	-0.28 (0.37)	-0.18 (0.41)
Range	-0.59 - 0.87	-0.80 - 1.32	-0.70 - 0.86	-0.80 - 1.32
**RANTES**				
N	33	16	29	78
Mean (SD)	-0.57 (0.03)	-0.13 (0.91)	-0.53 (0.07)	-0.47 (0.44)
Range	-0.62 - -0.52	-0.59 - 2.02	-0.60 - -0.23	-0.62 - 2.02
**IFNγ**				
N	33	16	29	78
Mean (SD)	-0.17 (0.38)	-0.06 (0.35)	0.04 (0.45)	-0.07 (0.41)
Range	-0.44 - 1.39	-0.40 - 0.75	-0.41 - 2.00	-0.44 - 2.00
**GM-CSF**				
N	33	16	29	78
Mean (SD)	-0.42 (0.10)	-0.38 (0.10)	-0.41 (0.09)	-0.41 (0.10)
Range	-0.62 - -0.15	-0.53 - -0.19	-0.53 - -0.19	-0.62 - -0.15
**TNFα**				
N	33	16	29	78
Mean (SD)	-0.34 (0.33)	-0.24 (0.25)	-0.18 (0.35)	-0.26 (0.33)
Range	-0.56 - 0.96	-0.54 - 0.34	-0.56 - 1.25	-0.56 - 1.25
**MIP-1β**				
N	33	16	29	78
Mean (SD)	-0.16 (0.86)	0.54 (0.79)	0.36 (0.74)	0.18 (0.85)

Range	-1.35 - 1.32	-0.80 - 2.31	-1.28 - 2.29	-1.35 - 2.31
**IFNα**				
N	33	16	29	78
Mean (SD)	-0.12 (0.07)	-0.18 (0.11)	-0.15 (0.10)	-0.14 (0.09)
Range	-0.23 - 0.07	-0.36 - -0.01	-0.30 - 0.12	-0.36 - 0.12
**MCP-1**				
N	33	16	29	78
Mean (SD)	0.69 (1.00)	1.22 (1.41)	1.83 (0.89)	1.22 (1.16)
Range	-0.62 - 2.65	-0.64 - 3.61	0.21 - 3.90	-0.64 - 3.90
**IL-9**				
N	33	16	29	78
Mean (SD)	0.13 (0.15)	-0.05 (0.29)	0.11 (0.19)	0.09 (0.21)
Range	-0.17 - 0.51	-0.67 - 0.36	-0.24 - 0.81	-0.67 - 0.81
**TNFβ**				
N	33	16	29	78
Mean (SD)	-0.02 (0.15)	-0.21 (0.24)	-0.11 (0.16)	-0.09 (0.19)
Range	-0.31 - 0.57	-0.76 - 0.08	-0.51 - 0.37	-0.76 - 0.57
**GROα**				
N	33	16	29	78
Mean (SD)	-0.33 (0.07)	-0.28 (0.22)	-0.33 (0.06)	-0.32 (0.11)
Range	-0.36 - 0.02	-0.37 - 0.53	-0.36 - -0.05	-0.37 - 0.53
**IL-1α**				
N	33	16	29	78
Mean (SD)	-0.17 (0.19)	-0.22 (0.33)	-0.30 (0.15)	-0.23 (0.22)
Range	-0.39 - 0.48	-0.50 - 0.81	-0.43 - 0.32	-0.50 - 0.81
**IL-23**				
N	33	16	29	78
Mean (SD)	0.25 (1.00)	-0.20 (0.20)	-0.27 (0.20)	-0.04 (0.71)
Range	-0.65 - 3.10	-0.57 - 0.10	-0.54 - 0.38	-0.65 - 3.10
**IL-15**				
N	33	16	29	78
Mean (SD)	0.02 (0.63)	-0.26 (0.05)	-0.17 (0.18)	-0.11 (0.44)
Range	-0.38 - 2.22	-0.34 - -0.13	-0.34 - 0.30	-0.38 - 2.22
**IL-18**				
N	33	16	29	78
Mean (SD)	-0.07 (0.45)	-0.02 (0.36)	0.04 (0.38)	-0.02 (0.41)
Range	-0.37 - 1.67	-0.37 - 0.80	-0.27 - 1.70	-0.37 - 1.70
**IL-21**				
N	33	16	29	78
Mean (SD)	-0.28 (0.29)	-0.14 (0.44)	-0.37 (0.19)	-0.28 (0.31)
Range	-0.63 - 0.75	-0.56 - 1.16	-0.63 - 0.32	-0.63 - 1.16
**IL-22**				
N	33	16	29	78
Mean (SD)	-0.00 (0.77)	-0.20 (0.28)	-0.27 (0.49)	-0.14 (0.60)
Range	-0.92 - 1.94	-0.54 - 0.56	-0.86 - 0.94	-0.92 - 1.94
**Amniotic fluid**				
	**AMN (N=34)**	**TPL (N=4)**	**CSR (N=29)**	**Total (N=67)**
**MIP-1α**				
N	34	4	29	67

Mean (SD)	0.59 (0.71)	0.40 (0.47)	-0.21 (0.30)	0.23 (0.67)
Range	-0.22 - 2.81	-0.23 - 0.89	-0.67 - 0.50	-0.67 - 2.81
**SDF-1α**				
N	34	4	29	67
Mean (SD)	0.32 (0.39)	1.25 (1.38)	0.11 (0.40)	0.28 (0.55)
Range	-0.30 - 1.21	-0.07 - 3.04	-0.59 - 1.51	-0.59 - 3.04
**IL-27**				
N	34	4	29	67
Mean (SD)	-0.16 (0.16)	-0.31 (0.03)	-0.37 (0.08)	-0.26 (0.16)
Range	-0.37 - 0.28	-0.34 - -0.27	-0.50 - -0.17	-0.50 - 0.28
**IL-1β**				
N	34	4	29	67
Mean (SD)	-0.31 (0.02)	0.88 (1.44)	-0.33 (0.02)	-0.25 (0.42)
Range	-0.33 - -0.27	-0.33 - 2.51	-0.35 - -0.27	-0.35 - 2.51
**IL-2**				
N	34	4	29	67
Mean (SD)	1.35 (1.42)	-0.16 (0.59)	0.56 (0.81)	0.92 (1.24)
Range	-0.46 - 5.14	-0.50 - 0.72	-0.42 - 3.48	-0.50 - 5.14
**IL-4**				
N	34	4	29	67
Mean (SD)	1.13 (1.44)	-0.13 (0.99)	1.32 (1.24)	1.14 (1.36)
Range	-0.56 - 5.40	-0.66 - 1.35	-0.38 - 4.51	-0.66 - 5.40
**IL-5**				
N	34	4	29	67
Mean (SD)	0.55 (1.17)	-0.04 (0.38)	-0.85 (0.54)	-0.09 (1.13)
Range	-0.95 - 3.41	-0.56 - 0.34	-1.41 - 1.25	-1.41 - 3.41
**IP-10**				
N	34	4	29	67
Mean (SD)	2.19 (1.39)	0.66 (2.23)	0.01 (0.70)	1.15 (1.60)
Range	0.87 - 6.98	-0.47 - 4.00	-0.49 - 3.00	-0.49 - 6.98
**IL-6**				
N	34	4	29	67
Mean (SD)	1.50 (1.71)	0.00 (0.61)	0.69 (1.79)	1.06 (1.75)
Range	-0.22 - 7.00	-0.31 - 0.92	-0.27 - 9.43	-0.31 - 9.43
**IL-7**				
N	34	4	29	67
Mean (SD)	0.54 (0.59)	0.25 (0.47)	-0.15 (0.45)	0.22 (0.62)
Range	-0.38 - 1.86	-0.30 - 0.66	-0.60 - 1.37	-0.60 - 1.86
**IL-8**				
N	34	4	29	67
Mean (SD)	0.41 (0.69)	1.82 (2.15)	-0.34 (0.38)	0.17 (0.91)
Range	-0.46 - 2.25	-0.55 - 3.94	-0.67 - 1.26	-0.67 - 3.94
**IL-10**				
N	34	4	29	67
Mean (SD)	0.99 (0.91)	0.30 (0.65)	-0.48 (0.84)	0.31 (1.11)
Range	-0.20 - 3.46	-0.41 - 1.16	-1.35 - 3.04	-1.35 - 3.46
**Eotaxin**				
N	34	4	29	67
Mean (SD)	-0.11 (0.36)	-0.43 (0.09)	-0.30 (0.32)	-0.21 (0.35)
Range	-0.47 - 1.60	-0.51 - -0.35	-0.60 - 1.08	-0.60 - 1.60

**IL-12 p70**				
N	34	4	29	67
Mean (SD)	-0.04 (0.35)	-0.34 (0.21)	-0.38 (0.14)	-0.21 (0.32)
Range	-0.45 - 0.92	-0.52 - -0.04	-0.61 - -0.17	-0.61 - 0.92
**IL-13**				
N	34	4	29	67
Mean (SD)	-0.24 (0.14)	-0.37 (0.10)	-0.31 (0.14)	-0.28 (0.14)
Range	-0.44 - 0.04	-0.49 - -0.27	-0.49 - -0.03	-0.49 - 0.04
**IL-17A**				
N	34	4	29	67
Mean (SD)	0.06 (0.37)	-0.21 (0.10)	-0.41 (0.10)	-0.16 (0.35)
Range	-0.30 - 1.20	-0.34 - -0.10	-0.54 - -0.12	-0.54 - 1.20
**IL-31**				
N	34	4	29	67
Mean (SD)	-0.21 (0.35)	0.19 (0.21)	-0.90 (0.34)	-0.48 (0.50)
Range	-0.79 - 0.71	-0.12 - 0.34	-1.37 - -0.12	-1.37 - 0.71
**IL-1RA**				
N	34	4	29	67
Mean (SD)	-0.69 (0.08)	0.38 (1.06)	-0.68 (0.20)	-0.62 (0.37)
Range	-0.78 - -0.43	-0.69 - 1.46	-0.79 - 0.16	-0.79 - 1.46
**RANTES**				
N	34	4	29	67
Mean (SD)	-0.59 (0.02)	-0.56 (0.02)	-0.47 (0.14)	-0.53 (0.11)
Range	-0.61 - -0.55	-0.59 - -0.53	-0.60 - 0.09	-0.61 - 0.09
**IFNγ**				
N	34	4	29	67
Mean (SD)	-0.39 (0.03)	-0.36 (0.06)	-0.40 (0.04)	-0.39 (0.04)
Range	-0.43 - -0.32	-0.42 - -0.28	-0.45 - -0.25	-0.45 - -0.25
**GM-CSF**				
N	34	4	29	67
Mean (SD)	0.58 (0.68)	-0.22 (0.31)	0.24 (0.81)	0.38 (0.75)
Range	-0.36 - 2.79	-0.45 - 0.23	-0.45 - 2.79	-0.45 - 2.79
**TNFα**				
N	34	4	29	67
Mean (SD)	0.41 (0.69)	-0.26 (0.30)	0.12 (0.44)	0.25 (0.60)
Range	-0.49 - 2.28	-0.49 - 0.19	-0.44 - 1.63	-0.49 - 2.28
**MIP-1β**				
N	34	4	29	67
Mean (SD)	0.43 (1.05)	1.29 (1.00)	0.49 (0.85)	0.51 (0.97)
Range	-0.87 - 4.41	0.64 - 2.78	-0.95 - 3.56	-0.95 - 4.41
**IFNα**				
N	34	4	29	67
Mean (SD)	-0.22 (0.14)	-0.09 (0.11)	-0.38 (0.06)	-0.28 (0.14)
Range	-0.38 - 0.40	-0.25 - 0.00	-0.49 - -0.26	-0.49 - 0.40
**MCP-1**				
N	34	4	29	67
Mean (SD)	0.16 (0.28)	0.81 (1.13)	-0.43 (0.30)	-0.06 (0.52)
Range	-0.28 - 0.79	-0.32 - 2.32	-0.64 - 0.64	-0.64 - 2.32
**IL-9**				
N	34	4	29	67

Mean (SD)	-0.18 (0.18)	0.22 (0.52)	-0.67 (0.11)	-0.37 (0.34)
Range	-0.47 - 0.21	-0.52 - 0.67	-0.84 - -0.33	-0.84 - 0.67
**TNFβ**				
N	34	4	29	67
Mean (SD)	-0.43 (0.15)	-0.12 (0.29)	-0.74 (0.15)	-0.55 (0.24)
Range	-0.66 - -0.14	-0.30 - 0.32	-0.88 - -0.38	-0.88 - 0.32
**GROα**				
N	34	4	29	67
Mean (SD)	0.35 (0.57)	0.13 (0.45)	0.20 (0.73)	0.27 (0.64)
Range	-0.21 - 2.68	-0.34 - 0.66	-0.36 - 3.13	-0.36 - 3.13
**IL-1α**				
N	34	4	29	67
Mean (SD)	-0.35 (0.06)	0.07 (0.45)	-0.23 (0.24)	-0.28 (0.22)
Range	-0.43 - -0.23	-0.38 - 0.62	-0.51 - 0.28	-0.51 - 0.62
**IL-23**				
N	34	4	29	67
Mean (SD)	-0.15 (0.18)	-0.31 (0.25)	-0.46 (0.26)	-0.29 (0.27)
Range	-0.46 - 0.24	-0.50 - 0.04	-0.85 - 0.35	-0.85 - 0.35
**IL-15**				
N	34	4	29	67
Mean (SD)	-0.17 (0.12)	-0.25 (0.04)	-0.31 (0.07)	-0.23 (0.12)
Range	-0.31 - 0.15	-0.30 - -0.19	-0.42 - -0.19	-0.42 - 0.15
**IL-18**				
N	34	4	29	67
Mean (SD)	-0.32 (0.11)	-0.30 (0.05)	-0.26 (0.13)	-0.30 (0.12)
Range	-0.41 - 0.17	-0.36 - -0.24	-0.42 - 0.01	-0.42 - 0.17
**IL-21**				
N	34	4	29	67
Mean (SD)	-0.30 (0.25)	-0.28 (0.45)	-0.72 (0.30)	-0.48 (0.35)
Range	-0.61 - 0.45	-0.80 - 0.28	-1.14 - -0.21	-1.14 - 0.45
**IL-22**				
N	34	4	29	67
Mean (SD)	-0.44 (0.32)	-0.31 (0.51)	-0.82 (0.46)	-0.60 (0.44)
Range	-1.05 - 0.04	-0.86 - 0.24	-1.57 - 0.04	-1.57 - 0.24

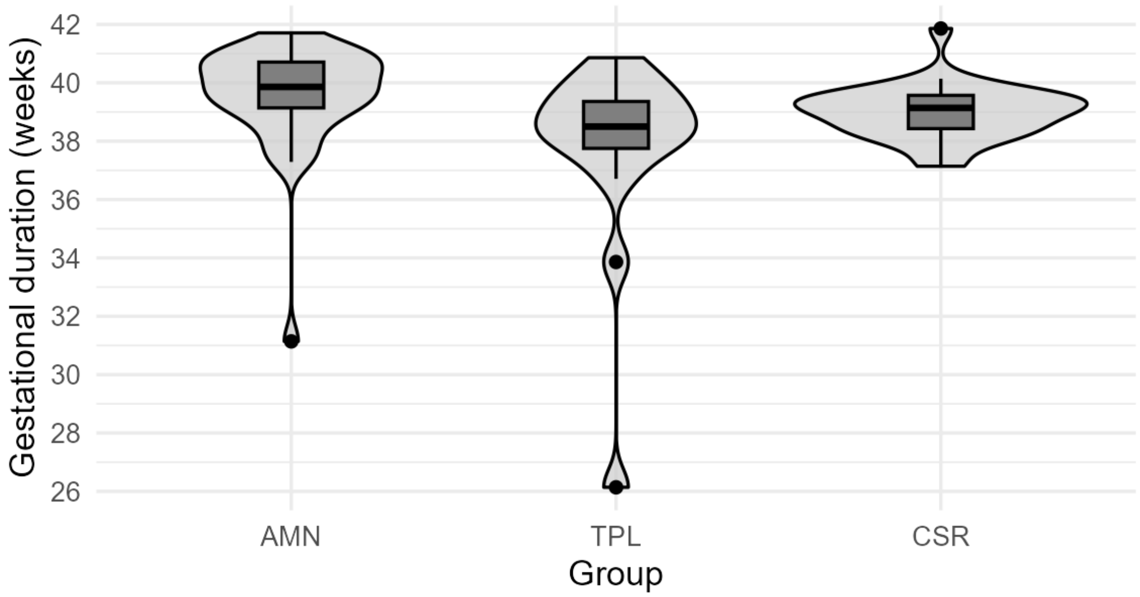

Figure S1: Violin plot with boxplot overlay showing the distribution of gestational duration across groups (AMN: amniocentesis; TPL: threatened preterm labor; CSR: cesarean).
